# Medical education through an invasion: insights from an elective programme for Ukrainian medical students at the University of Cambridge

**DOI:** 10.3389/fmed.2023.1211526

**Published:** 2023-09-28

**Authors:** Joy Hodkinson, Mark Lillicrap, Paul Wilkinson, Stephen Bevan, Tamari Shenheliia, Vilena Chupina, Jonathan Fuld

**Affiliations:** ^1^NHS Lothian, University of Edinburgh, Edinburgh, United Kingdom; ^2^School of Clinical Medicine, University of Cambridge, Cambridge, United Kingdom

**Keywords:** Ukraine, Kharkiv, Cambridge, medical, student, war, placement, elective

## Abstract

Medical students in Ukraine have faced extraordinary disruption to their clinical studies with both the COVID-19 pandemic and subsequent Russian military invasion forcing a majority of their learning to be conducted remotely. Over the summer of 2022, the School of Clinical Medicine, University of Cambridge hosted 20 medical students from Kharkiv National Medical University for a seven-week intensive clinical elective programme. The aim was to provide an immersive clinical placement that would help students to attain the necessary knowledge and experience to become competent and confident practising doctors. This perspective piece aims to support the development of future equivalent exchanges through outlining the placement’s context, its planning and implementation, evidence of placement impact, and finally reflections and learning points.

## 1. Introduction

**Figure 1:**
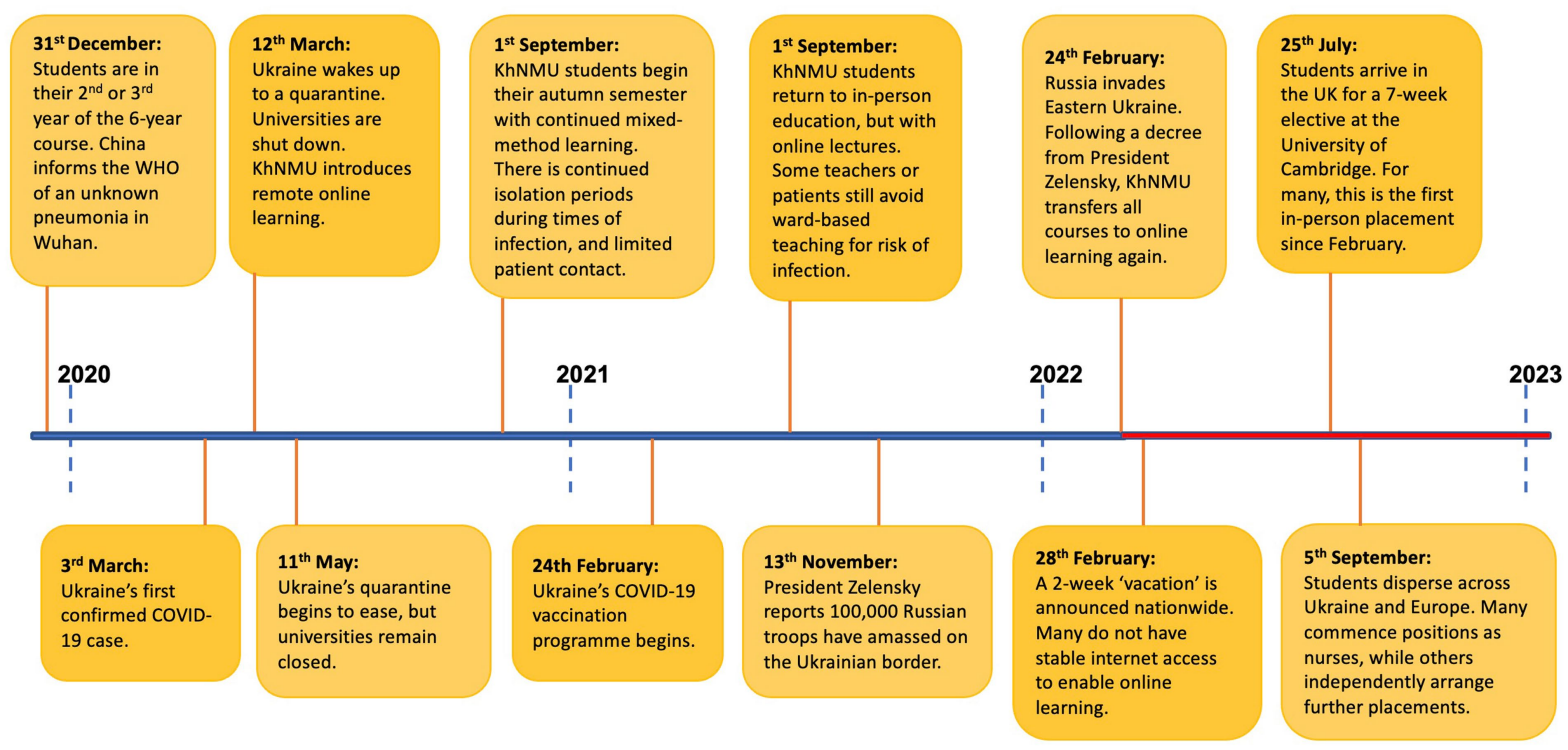
Timeline outlining the disruption that students in the class of 2017 and 2018 at Kharkiv National Medical University have experienced over the course of the last 3 years (1, 2).

The wish to support our counterparts in Ukraine following the Russian invasion was a sentiment echoed across the UK and its medical schools. Dialogue between UK medical schools in March 2022 established what response would bring most value. While Ukrainian medical schools were delivering theoretical teaching online, the major educational omission was clinical placements, which were forced to be halted (Figure 1). The hope therefore was for UK medical schools to help to bridge this gap by providing hospital placements to Ukrainian medical students.

A system of twinning was established between Ukrainian and UK medical schools. This would enable cohorts to remain together, facilitate administration, and allow placements to be tailored to redress each university’s most impacted curricular components. Thus, Cambridge School of Clinical Medicine and Kharkiv National Medical University (KhNMU), situated in Ukraine’s second city close to the frontline of the initial invasion, were paired. This process was facilitated by Cormack Consultancy Group (3) and the Medical Schools Council (1).

## 2. Placement arrangement and educational design

### 2.1. Logistics

It was agreed that placements would occur over the summer months. This timing facilitated the sourcing of supervisors, accommodation, and ensured minimal competition with local students for clinical opportunities. Weekly meetings between Cambridge University Hospitals (CUH), the Clinical School and colleagues at KhNMU drove placement development forward. Standing items included student selection, funding, visas, travel, accommodation, educational content, welfare, and communications. Smaller working groups reported back to weekly meetings which ran from late May–July 2022.

### 2.2. Selection of students

Electives were planned for up to 30 students. To be eligible, students were required to be Ukrainian Nationals proficient in the English language with CEFR scores of B2 or above. Change to Ukrainian Government Policy in June 2022 enabled male students to attend, following Government approval. Given the late timing of this decision, most students were female. Within these guidelines, KhNMU then invited students in their 4th and 5th years (at the time of selection) of their six-year medical degree to apply via means of an online application form. Students were subsequently ranked according to a combined score that was based upon their language proficiency, academic performance, and participation in scientific work, each with equal weighting.

### 2.3. Funding, hospitality and support

Funding for the programme was by virtue of two generous donations; one from Illumina (4), and another anonymous donation specifically to cover accommodation expenses. The total cost of the programme delivered to 20 students was £64,085. As such, the cost of visas, travel, accommodation, food, and other essentials were covered. Students resided in Homerton College, Cambridge, a 10-min walk from the hospital, with catering and kitchen facilities, and a library. Students additionally received a stipend of £500. For each of their three hospital placements, students were allocated a senior doctor working within the specialty to act as a supervisor. Junior doctor mentors, as outlined below, also provided guidance, particularly relating to the clinical environment. Students were encouraged to utilise KhNMU’s 24/7 mental health helpline should they require support. However, contact details for Cambridge Clinical School’s Welfare Officers were also provided.

### 2.4. Educational planning

To provide an educationally beneficial experience, the learning needs of the students were discussed with KhNMU. Situated clinical learning (5) was identified as the key aim. While KhNMU faculty had successfully delivered online teaching covering core topics relating to professional knowledge (6), students had not applied this through active participation within the clinical environment, nor received in-person clinical supervision. They therefore had not been able to consistently develop the professional skills expected of a medical graduate. The elective therefore comprised structured clinical placements based in Cambridge University Hospitals, the Royal Papworth Hospital, and the Ida Darwin Hospital.

### 2.5. Introductory week

**Figure 2:**
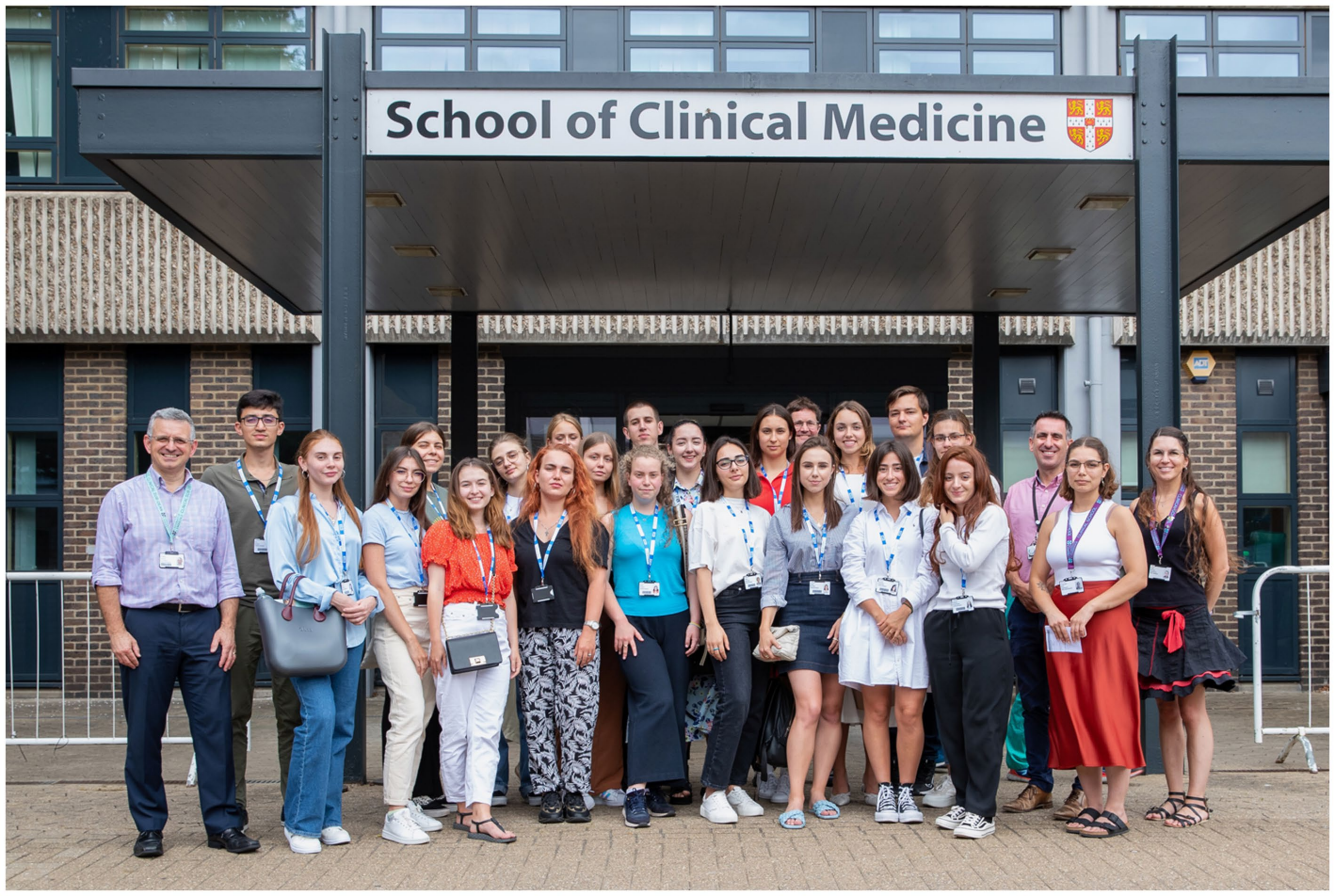
Students from Kharkiv National Medical University are welcomed to Cambridge School of Clinical Medicine and begin a week of inductions prior to commencing their 6-week clinical placement. Permissions: University of Cambridge.

The introductory week outlined the structure of the elective and incorporated mandatory NHS training and occupational health assessments (Figure 2). Administrative tasks were nestled between clinical learning, including practical skills training and basic life support. Given the students’ lack of patient-facing experiences, a bespoke communications skills session was organised, based upon the Cambridge-Calgary Model (7) and incorporating simulated clinical encounters. Larger group sessions were arranged outlining the NHS structure and medical students’ professional responsibilities as per GMC guidance (8). Two half-day seminars took place: developing clinical reasoning skills and principles of palliative care.

### 2.6. Clinical rotations

Students were subsequently divided into three groups and entered two-week clinical rotations in medicine, surgery, and a student selected component (SSC). There were no pre-requisite selection criteria for the 12 available SCC options, and all students received their first-choice SSC placement.

Weekly hourly seminars were delivered by faculty and covered common clinical scenarios. Aside from this, didactic teaching was minimised, given its suitability for remote formats. Instead, weekly one-hour, near-peer small-group bedside teaching was delivered by junior doctors recruited from CUH. Bedside teaching focused upon adult medical and surgical history and examinations, diagnostic reasoning, interpretation of investigations and management planning, addressing core intended learning outcomes including GMC outcomes 10, 11, 12 and 14 around interpersonal skills, diagnosis and medical management (6). The neer-peer sessions were structured so that the junior doctors met the same 5–6 students each week, and thus could tailor sessions to meet students’ specific learning objectives. They were guided to cover adult medical and surgical assessment routines using a thematic approach with cardiovascular, respiratory, abdominal (medical/surgical), neurological and musculoskeletal focus to the sessions over the 6 weeks. Generally, these sessions took place at the bedside, on the wards of CUH, with review of assessment routines, confirmation of clinical signs and discussion of relevant clinical reasoning (including diagnosis, investigations, management, etc.). Furthermore, the junior doctors undertook a teaching-to-teach training session prior to the elective that included briefing on the details of the programme, an overview of the Kharkiv curriculum, the intended learning outcome for the weekly sessions and the support processes that were in place. One-to-one debriefs with each student took place in the final week to reflect upon student’s developing professional knowledge, skills and behaviours. Pro-forma similar to that used for University of Cambridge medical students aided reflection. These debriefs provided formative feedback that was included in the student’s learning portfolio.

### 2.7. Student feedback

Students completed end-of-placement feedback, to review the impact of the programme. Thirteen of the twenty students completed the paper form that was distributed to students on conclusion of the placement. An informal group interview was also conducted with students. Additionally, one student and one consultant clinical supervisor were interviewed individually. Upon conclusion of the programme, the Institute of the Quality of Education at KhNMU developed a short paper questionnaire to further measure placement impact. The above evidence underpins the following programme reflections and discussion.

## 3. Student satisfaction and placement reflections

### 3.1. Clinical communication skills (CCS)

*“It’s been hard, as online we’ve had minimal practise. My Kharkiv study group has the best academic marks but we struggle with patient interaction. After this workshop, my fear is gone.”*

Simulated communication stations, a mainstay of medical education in the UK, have not been a component of the curriculum at KhNMU. Feedback resoundingly affirmed the inclusion of these sessions. Timing the workshops towards the beginning of the seven-weeks enabled students to further hone their skills. Interestingly, students spoke of the differences in communication styles between medical professionals in the United Kingdom and Ukraine. In particular, students were impressed by the culture of shared-decision making between doctors and patients in the NHS, and it was evident that they had reflected upon the distinct benefits and challenges of the differing cultures of communication. Encouragingly, students planned to adopt the format of the session and establish a student-led communications skills workshop to share their learning points with KhNMU peers. KhNMU faculty members have subsequently expressed enthusiasm for adopting such sessions.

### 3.2. Varied learning opportunities

*“Some people know from childhood that they want to be a surgeon, but I never did so I would say ‘I’m not a surgical person’. I never had the chance to observe in Theatre but when I finally watched open heart surgery I thought ‘yes, I could do this’.”*

The inclusion of a SSC was a highlight for students. Varied specialty options were available. These ranged from child and adolescent psychiatry to transplant surgery. KhNMU’s curriculum had necessarily honed upon core topics, and the chance to explore interests was limited. Surgery was particularly popular amongst students, many of whom had never had the chance to scrub into Theatre. Indeed, one consultant ENT surgeon shared that “the eagerness of the Ukrainian students to learn put our UK medical students to shame.”

Outside of SSCs, students were welcoming of the flexibility in their schedules, and of the chance to pursue their interests. Although importantly a small number of students expressed concerns of feeling “lost”, unaccustomed to independently seeking learning opportunities. The inclusion of junior doctor led bedside teaching was well received, ensuring continuity as well as allowing pastoral support alongside teaching delivery. Student feedback referenced increased confidence in their clinical skills.

### 3.3. CUH healthcare infrastructure

*“We have similar [IT] in Ukraine, but it’s nowhere near as good. It’s amazing to have patient’s records accessible at your fingertips.”*

Without doubt it was “EPIC”, CUH’s clinical software, that elicited the most rejoicing amongst students. Students expressed their desire to push towards the future development of improved IT programmes in Ukraine.

Another clear highlight was access to the Deakin Centre, a 24/7 simulated clinical learning environment where students could practise cannulation and catheterisation to their hearts’ content. Such practical elements were understandingly popular given the obvious limitations of online learning environments.

### 3.4. Student concerns

The timing of the placement during summer months conferred the drawback of coinciding with periods of staff annual leave. This meant that on occasion students received less support from the senior clinicians who were assigned to supervise their placements, and some teaching opportunities did not materialise. Students emphasised that on such occasions, healthcare staff endeavored to provide them with alternative opportunities, but the usefulness was variable.

Importantly, one student raised concerns regarding sexism on placement, particularly highlighting Theatre as a rife environment with only male students actively involved and encouraged to assist on some occasions. Their experiences point towards an ongoing need for active initiatives to tackle sexism within the NHS and echo experiences of UK female surgeons (9).

### 3.5. Contrasting healthcare systems

*“In Ukraine surgeons are ‘Gods’ and you are expected to know everything. But in the UK we could ask simple questions. They were on our side.”*

It was encouraging to hear students reflect, with great insight, into the UK’s healthcare delivery. Students were grateful for the openness of staff to their queries, and for the safe learning environment that was fostered. One student recalled the humility that UK doctors demonstrated, greatly impressed by a consultant offering that they did not know the answer to a question.

Students also considered the benefit to patients of Ukraine’s more accessible primary care system, with GPs contactable over text at any time of day, while valuing the UK’s emphasis on promoting doctors’ work-life balance. Indeed, students spoke of the aspects of their education in Ukraine that were “definitely better” and sung praises of KhNMU.

Finally, it is worthy of note that the programme has had the supplementary benefit of furthering the English language skills of students, as noted by KhNMU faculty. This is a particular advantage given the need for many to continue their studies overseas.

## 4. Discussion

Medical students studying in Ukraine have faced an unthinkable level of disruption to their studies. As may be seen in the above timeline, some students have had only a few months of their studies unencumbered from the COVID-19 pandemic and the war in Ukraine. In our current era much may be gained from online learning. Yet it is without question that one cannot become a safe, competent, and confident doctor without in-person experiential learning. The aim of the placement therefore was to provide students with a seven-week period in which they could immerse themselves in the clinical environment, free from the toll of wartime, financial burdens, or the administrative challenges of seeking out educational opportunities. In the following discussion we consider the overall impact of the placement, and explore how future placements at UK medical schools might build upon our experiences.

### 4.1. Placement impact

*“The pandemic, the war, and endless online learning had left me close to burnout. This summer’s placement came at just the right time and prevented this. I now can’t wait to become a doctor.”*

Student feedback was unanimous in the view that the seven weeks had strengthened their communication skills, practical and examination skills, and clinical acumen. Indeed, KhNMU faculty have been impressed by students’ tangible academic progress, enabling them to be signed off for specific components of their curriculum. The enthusiasm expressed by KhNMU faculty to incorporate elements of the elective, notably the CCS components, is testament to its beneficial impact for students, many of whom continue to have minimal patient interactions.

Due to the ongoing conflict, after the placement, students returned to Ukraine, and across Europe. There they commenced their 5th and 6th years of their degree, comprising a combination of KhNMU-led distance learning and time spent either in temporary employment as a nurse, or in self-organised and self-funded placements. Whilst the elective placement has clearly bolstered students’ clinical education, it would be naïve to suggest that a seven-week elective can make amends for verging on three years of educational disruption. As such, no students have yet been granted an early graduation from KhNMU, although we hope that their experience will be formally recognised and facilitate their timely progression to working as doctors in Ukraine.

### 4.2. Shaping career paths

In contrast to the United Kingdom, upon graduation from medical school in Ukraine, students must choose a speciality to pursue. For many students involved in the exchange, their experience of clinical medicine has been limited to a few specialities. The prospect of their career paths being sculpted by such limited exposure is understandably daunting. The programme has undoubtedly broadened their clinical experience, particularly in the area of surgery. Future programmes should continue to prioritise flexibility and student choice within their structure so as to capture this valuable element.

### 4.3. Strengthening Ukraine’s medical workforce

*“I really want to return to Ukraine and contribute what I have learnt. I think if the war stops, 90% of students will go back as soon as possible. It is home.”*

Electives have long been a core component of UK medical courses, with good reason. The chance to encounter varied healthcare delivery confers cross-cultural learning. Students’ perceptive feedback demonstrated that this knowledge exchange had indeed taken place. As organisers of the exchange, we reflected upon the potential consequence of students being encouraged to pursue careers elsewhere than Ukraine. However, this was resoundingly not the case. The NHS was by no means a utopia in their eyes. Overwhelmingly, students echoed a desire to return “home” with haste, and to contribute to the rebuilding of their nation post-conflict.

### 4.4. Learning points

There were numerous logistical hurdles to the development of placements at relatively short notice. Crucially, the programme did not occur without significant investment, and was reliant upon the generosity of donors. We are mindful that continued financial backing is a prerequisite to future initiatives. Furthermore, the administrative time in arranging the programme was substantial, and it would be appropriate to fund the creation of a specific administrative post.

A barrier was represented by the requirement for students to obtain visas, which was made challenging by the absence of a British consulate in Ukraine. It would be sensible to create an exchange waiting list, to maximise the number of attending students should visa issues arise. Future initiatives should factor in delays in obtaining visas into timelines, as well as advise students of visa application processes.

Occupational health assessment, specifically TB screening, was problematic for students, some of whom were required to undergo multiple screenings at different international borders. Again, a waiting list might be wise, given the possibility of students testing positive. Consideration of ways to minimise such bureaucratic and financial burden students forced to cross multiple borders should occur.

Although the faculty teams in Kharkiv and Cambridge worked together closely, to ensure that the programme developed met the needs of the Kharkiv students, the intended learning outcomes of the programme and the framework developed was based on the outcomes and curriculum used in Cambridge and mapped to the GMC outcomes for graduates. There was an exchange of information, across both faculty teams, to ensure that the programme that was developed explicitly linked with prior student learning (delivered by the Kharkiv faculty). However, for future programmes and to support educational quality assurance processes, it would be worthwhile more explicitly aligning the intended outcomes of the programme with those of the overall Kharkiv programme, since this would allow students and faculty (in both institutions) to review student learning from the programme and map the learning to future assessment processes (10, 11).

Feedback from students has focussed our attention upon the difficulty that reduced hospital staffing levels in summer presents. The timing of the placement was considered carefully, and it was deemed infeasible to accommodate the students during the academic year. In future, the roles and expectations for senior clinical supervisors should be made clear in advance of volunteering. Additionally, enlisting a larger volunteer pool, to cover unexpected absences, would be prudent. Notably, the inclusion of near-peer teaching with junior doctors helped to provide students with continuity and mentorship and acted as an antidote to some of the aforementioned difficulties. We would encourage the incorporation of this element into future programmes, as it was greatly valued.

Finally, whilst Cambridge stipulated some eligibility criteria, KhNMU selected academically higher achieving students. The aim of the programme was to help ameliorate some of the disruption to training imposed by the pandemic and war, and this distinction does not necessarily align with the objective. Given the level of financial support provided, the need to instead prioritise students from lower-income backgrounds should be considered since they may not otherwise be afforded overseas placements. As such, we need to think carefully about future selection criteria.

### 4.5. Future directions

There is a mutual wish to repeat the programme, and ongoing dialogue to arrange an exchange in summer 2023. This commitment is regardless of the exact political context, as medical students will undoubtedly still benefit from additional clinical exposure, given the continued disruptions. We also hope to increase the intake by 50%, up to a total of 30 students. Impetus is needed to secure funding, which represents the main obstacle.

Our aim, ultimately, is that this placement could enable students to begin their working careers sooner, so as to bolster Ukraine’s medical workforce (as has occurred in the NHS during the COVID-19 pandemic). As the current cohort progresses through their training, it remains to be seen whether this will be possible, and we await further impact statements from KhNMU.

Future programmes should consider the changing needs of students as the war, and thus medical education, in Ukraine evolves. The focus and content of placements, and specialities experienced, may need to be adapted, as new cohorts progress through training with varying curricular gaps. We recognise our role to primarily be that of hosts and facilitators, and as such are keen to be guided by KhNMU faculty and Ukraine’s medical student body in this regard.

Multiple UK-Ukraine Medical School twinnings took place in 2022, and several other medical schools have supported Ukrainian students by means of clinical placement, or have expressed an interest in doing so. Ukraine has 45 medical schools accredited by the World Health Organisation (WHO) and UNESCO (12) and the conflict shows no sign of imminent resolution. As such, we are eager to share our learnings, with the aspiration to support the establishment of equivalent programmes. We are mindful to recognise that many medical students across the globe are impacted by ongoing conflicts, and other such barriers to their education. While our donors specifically gifted funds for Ukraine, we hope that the positive impact of this exchange will encourage support for medical students who are facing adversity across the globe.

## 5. Conclusion

The positive experience of the Ukrainian medical students is testament to the effectiveness of institutional collaboration, and highlights ways in which UK medical schools may support our peers in Ukraine. We asked students if there was anything that they might want readers to take away from this article. Without hesitation their response was simply that of thanks to those who have facilitated the programme.

The fortitude of the Ukrainian students, who display such drive to continue their medical education, must be acknowledged. These are a cohort of medical students who have, and continue, to study under circumstances that are unimaginably challenging for many. Their dedication and enthusiasm to further their education concurrent to the ongoing conflict is admirable. We are hopeful that the exchange has offered some reprieve from the disruption to their studies and respite from the grave weight of wartime.

## Data availability statement

The raw data supporting the conclusions of this article will be made available by the authors, without undue reservation.

## Ethics statement

Written informed consent was obtained from the individual(s) for the publication of any potentially identifiable images or data included in this article.

## Author contributions

JF, PW, and ML contributed to the development and running of the elective programme along with Cambridge University Hospitals and Cambridge School of Clinical Medicine staff. JH and JF contributed to the conception of the manuscript structure and content. JH wrote the draft of the manuscript, conducted interviews with students, and produced figures. PW contributed to writing the introduction while ML contributed to writing the educational planning, introductory week, and clinical rotations. SB facilitated student interviews and provided figures. TS contributed to the development of the timeline figure. VC contributed to the student perspective quotes used in the manuscript. Further drafts, revisions and edits of the manuscript were conducted by JH and JF. All authors contributed to the article and approved the submitted version.
